# Comparative and phylogenetic analyses of Loranthaceae plastomes provide insights into the evolutionary trajectories of plastome degradation in hemiparasitic plants

**DOI:** 10.1186/s12870-024-05094-5

**Published:** 2024-05-16

**Authors:** Lilei Tang, Tinglu Wang, Luxiao Hou, Guangfei Zhang, Min Deng, Xiaorong Guo, Yunheng Ji

**Affiliations:** 1 Key Laboratory of Phytochemistry and Natural Medicines, Kunming Institute of Botany, Chinese Academy of Sciences, Kunming, 650201 China; 2 School of Traditional Chinese Medicine, Guangdong Pharmaceutical University, Guangzhou, 510006 China; 3 Kunming College of Life Science, University of Chinese Academy of Sciences, Kunming, 650201 China; 4 School of Ecology and Environmental Science, Yunnan University, Kunming, Yunnan 650504 China; 5 Yunnan Key Laboratory of Plant Reproductive Adaptation and Evolutionary Ecology and Institute of Biodiversity, Yunnan University, Kunming, Yunnan 650504 China

**Keywords:** Facultative parasitism, Gene loss, Obligate parasitism, Plastome degradation, Pseudogenization

## Abstract

**Background:**

The lifestyle transition from autotrophy to heterotrophy often leads to extensive degradation of plastomes in parasitic plants, while the evolutionary trajectories of plastome degradation associated with parasitism in hemiparasitic plants remain poorly understood. In this study, phylogeny-oriented comparative analyses were conducted to investigate whether obligate Loranthaceae stem-parasites experienced higher degrees of plastome degradation than closely related facultative root-parasites and to explore the potential evolutionary events that triggered the ‘domino effect’ in plastome degradation of hemiparasitic plants.

**Results:**

Through phylogeny-oriented comparative analyses, the results indicate that Loranthaceae hemiparasites have undergone varying degrees of plastome degradation as they evolved towards a heterotrophic lifestyle. Compared to closely related facultative root-parasites, all obligate stem-parasites exhibited an elevated degree plastome degradation, characterized by increased downsizing, gene loss, and pseudogenization, thereby providing empirical evidence supporting the theoretical expectation that evolution from facultative parasitism to obligate parasitism may result in a higher degree of plastome degradation in hemiparasites. Along with infra-familial divergence in Loranthaceae, several lineage-specific gene loss/pseudogenization events occurred at deep nodes, whereas further independent gene loss/pseudogenization events were observed in shallow branches.

**Conclusions:**

The findings suggest that in addition to the increasing levels of nutritional reliance on host plants, cladogenesis can be considered as another pivotal evolutionary event triggering the ‘domino effect’ in plastome degradation of hemiparasitic plants. These findings provide new insights into the evolutionary trajectory of plastome degradation in hemiparasitic plants.

**Supplementary Information:**

The online version contains supplementary material available at 10.1186/s12870-024-05094-5.

## Background

Parasitic plants rely on their host to alleviated competition with other plant species for essential resources (e.g., light, water, soil nutrients), thereby enhancing their adaptive capacity in unfavorable environmental conditions; consequently, parasitism is generally perceived as a life strategy that evolves under natural selection and environmental pressures [1]. To date, approximately 4,750 parasitic species have been documented in angiosperms and it is estimated that the lifestyle transition from photosynthetic autotrophy to parasitism may have independently evolved at least 12 or 13 times in the angiosperm tree of life [1, 2]. Based on their photosynthetic capacity, parasitic plants are divided into two categories: hemiparasites and holoparasites [3–5]. Hemiparasites retain varying degrees of photosynthetic capacity while obtaining nutrients (primarily water and mineral elements) from their hosts. Contrarily, holoparasites exhaustively lose their photosynthetic capacity, with severely or entirely degraded leaves; therefore, they must completely rely on the host plants for energy and nutrients [3, 5, 6].

Chloroplasts, which originated from the endosymbiosis of photosynthetic autotrophic cyanobacteria within primitive eukaryotic cells, serve as vital centers for energy acquisition and carbohydrate production in plants [7, 8]. Due to their distinct origin, chloroplasts possess autonomous genomes and transcription/translation systems that synergistically interact with the nuclear genomes for plastid protein synthesis [8, 9]. Although the chloroplast genome (plastome) is highly conserved in most photosynthetic autotrophic angiosperms in terms of genome size, structure, and gene content, the lifestyle transition from autotrophy to heterotrophy might relax the purifying selection pressures that play a crucial role in maintaining the stability of plastomes [10], resulting in varying degrees of gene loss, pseudogenization, size reduction and structural rearrangement in parasitic plastomes [10–16]. Previous studies have demonstrated that different levels of reliance on host plants for energy and nutrient requirement can exert various intensities of selective pressures on the plastomes of parasitic plants; as a result, holoparasites exhibit higher levels of plastome degradation compared to their hemiparasitic relatives [13–19].

Hemiparasitic plants are further classified into facultative root-parasites and obligate stem-parasites based on their feeding modes and nutritional dependence on the hosts [20]. Considering the relatively higher degrees of trophic reliance of obligate stem-parasites on their hosts [21], purifying selection of plastid genes tends to be gradually relaxed during the transition from facultative parasitism to obligate parasitism. Therefore, obligate stem-hemiparasites are expected to have higher levels of plastome degradation than facultative root-hemiparasites [14, 22]. Surprisingly, this theoretical prediction has not yet been empirically validated, as previous comparative studies on the plastomes of Santalales hemiparasites revealed that obligate stem-parasites did not consistently exhibit a higher level of gene loss or pseudogenization than facultative root-parasites [23–25,]. Additionally, a previous study suggested that plastome degradation in hemiparasitic plants may follow a ‘domino effect’, where initial losses of plastid genes trigger chain reactions leading to subsequent gene losses [23]. However, the evolutionary mechanisms underlying the enigmatic ‘domino effect’ of plastome degradation in hemiparasitic plants remain elusive. These unresolved issues leave critical gaps in the exploration of evolutionary trajectories of plastome degradation in hemiparasitic plants.

Previous studies have demonstrated multiple independent evolutionary transitions from facultative root-parasitism to obligate stem-parasitism in Santalales [1, 24, 25], and proposed that plastome degradation in Santalales hemiparasites may have evolved in a lineage-specific manner [24–26]. This suggests that the degree of plastome degradation may exhibit substantial variation among phylogenetically distant facultative root-parasites, potentially surpassing the differences in plastome degradation observed between closely related root-parasites and obligate stem-parasites. Consequently, previous studies [24, 25, 27] on the retrogressive evolution of hemiparasitic Santalales plastomes may be flawed, as comparative analyses of plastomes based solely on lifestyle variations (facultative root-parasitism vs. obligate stem-parasitism) without considering phylogenetic relationships could lead to biased inferences.

The hemiparasitic lineage Loranthaceae, which includes approximately 76 genera and more than 1,000 species, is the largest family within Santalales [20, 28, 29]. Within Loranthaceae, three monotypic genera, *Nuytsia*, *Atkinsonia*, and *Gaiadendron*, are facultative root-parasites, while the remaining genera are obligate stem-parasites [20]. Given the monophyletic origin of obligate stem-parasitism from facultative root-parasitism in Loranthaceae [20, 29, 30], this family provides an ideal system to investigate whether obligate stem-parasites have a higher degree of plastome degradation than closely related facultative root-parasites. To gain a better understanding of the evolutionary trajectories of plastome degradation in hemiparastic plants, this study aims to (1) verify the theoretical prediction that the lifestyle transition from facultative root-parasitism to obligate stem-parasitism may lead to further degradations of hemiparasitic plastomes [14, 27] and (2) explore the plausible events that could have triggered the ‘domino effect’ in the plastome degradation among Loranthaceae hemiparasites. To achieve these objectives, we sequenced the plastomes of 22 Loranthaceae hemiparasites and combined them with publicly available data, yielding an extensive dataset comprising the plastomes of 48 species from 14 genera within this family. Under the framework of plastome phylogeny, we analyzed plastome degradation in each clade to determine whether there was lineage-specific gene loss, pseudogenization, or plastome structural variation within Loranthaceae. Additionally, we compared the plastome features of obligate stem-parasites with those of facultative root-parasites to explore differences in the degree of plastome degradation.

## Materials and methods

### Plant materials, illumina sequencing, plastome assembly and annotation

In total, 22 species from eight genera of Loranthaceae were collected from the wild (voucher specimens were deposited in the herbarium of Yunnan University and taxonomically identified by Dr. Min Deng), within voucher information being presented in Table S1. The genomic DNA of each sample was extracted from approximately 100 mg of silica gel-dried leaf tissues using a modified CTAB method [31]. The purified genomic DNA was use to prepare a paired-end Illumina sequencing library (with an average insert size of approximately 350 bp) for each sample, employing the TruSeq DNA Sample Prep Kit (Illumina, Inc., San Diego, CA, USA) following the manufacturer’s guidelines. Low-coverage genome sequencing was performed on an Illumina HiSeq 2500 platform to generate approximately four Gbp raw reads for each sample.

Raw Illumina reads were filtered using an NGS QC tool kit [32] to remove adaptors and reads with ambiguous bases. The complete plastome of each sample was *de novo* assembled using NOVOPlasty v.2.7.0 [33] with the k-mer size set at 31, and the large subunit of the RuBisCO gene (*rbcL*) of *Taxillus sutchuenensis* (MG999457) was utilized as the seed for plastome assembly. These assembled plastomes were annotated using PGA [34], and the boundaries of start/stop codons and introns/extrons of protein-coding genes were checked using Geneious v10.2 [35]. The tRNAscan-SE 1.21 [36]. was used to verify the transfer RNA (tRNA) genes using default parameters. All annotated plastomes were deposited in the NCBI GenBank database and their accession numbers are listed in Table S1. In addition to these newly sequenced plastomes, publicly available complete plastomes of 26 Loranthaceae species (Table S2) were obtained from the NCBI GenBank database for phylogenetic and comparative analyses. These publicly available plastomes were reannotated using the same method.

### Phylogenetic analyses

In total, 48 species of Loranthaceae hemiparasites were sampled for phylogenetic analysis. *Erythropalum scandens* (Erythropalaceae), an autotrophic relative of Loranthaceae [1, 28, 30] was selected as the outgroup. Based on the plastome dataset, 55 plastid protein-coding genes (PCGs) commonly shared by these species were extracted from each plastome using Geneious v10.2 [35]. These PCGs were aligned separately using MAFFT v7.3.1 [37], and each CDS matrix was concatenated into a supermatrix (3,8153 bp) using Phylosuite [38]. Based on the concatenated matrix, maximum likelihood (ML) and Bayesian inference (BI) approaches were employed to infer the intrafamilial phylogenetic relationships of Loranthaceae. ML analysis was implemented by IQ-TREE V2.1.2 [39, 40] using UFBoot2 with the best-fit model TVM + F + R2 determined by Modelfinder [41]; 1,000 replications were adopted to calculate the standard bootstrap percentage for each node. MrBayes v3.2.7a [42] was used for the BI analysis. According to the Akaike information criterion (AIC), the best-fitting partitioning schemes and models were selected for each gene with default values using PartitionFinder v2.1.1 [43]. . The Markov chain Monte Carlo algorithm was run for five million generations with every 5,000 generations for tree sampling. Trees resulting from the first 25% of generations were discarded as “burn-in”, and the remaining trees were used to build the 50% majority-rule consensus tree and estimate the posterior probability values (PP). FigTree 1.4.6 [44] was used to present and edit the phylogenetic trees.

### Comparative analyses of Loranthaceae plastomes

Genomic features, including genome size, gene content, and GC percentage of the whole plastome, LSC regions, SSC regions, and IR regions, were compared according to the sequence and annotation using Geneious v10.2 [35]. The boundaries of LSC, SSC, and IRs of each plastome were compared to check IR expansion/contraction in Loranthaceae plastomes using Geneious v10.2 [35] by comparison with their autotrophic relative, *Erythropalum scandens*. The microsyntenic structure of Loranthaceae plastomes was investigated using the multiple genome alignment tool, Mauve v2.3.1 [45], after removing one copy of the IR region for each plastome. Gene loss and pseudogenization events detected in each plastome were mapped to a phylogenetic tree to trace the evolutionary trajectory of plastome degradation associated with the evolution of the hemiparasitic lifestyle in Loranthaceae.

## Results

### Plastome features of Loranthaceae hemiparasites

The plastomes of these 48 Loranthaceae hemiparasites exhibited a typical quadripartite structure, consisting of a pair of inverted repeat regions (IRs, 20,118–26,801 bp) separated by a large single copy (LSC, 67,837–79,519 bp) and a small single copy (SSC, 5,059–7,719 bp) regions (Table 1). The lengths of these plastomes varied from 115,635 bp (*Dendrophthoe pentandra*) to 139,027 bp (*Nuytsia floribunda*), exhibiting a genomic size difference of 24,004 bp. The guanine and cytosine (GC) content of these Loranthaceae hemiparasites ranged from 35.9 to 37.8% (Table 1), and exhibited variations across the four regions. Specifically, the IR regions displayed the highest GC content (42.0–43.0%), followed by the LSC region (33.4–35.5%) and the SSC region (24.1–26.9%) (Table 1). The plastome of the autotrophic *Erythropalum scandens* harbored a total of 114 genes, comprising 79 protein-coding genes (PCGs), 30 tRNAs, four rRNAs, and one ycf15 pseudogene. In contrast, the plastomes of Loranthaceae species exhibited varying degrees of gene loss and pseudogenization, encompassing 92–100 plastid genes including 59–66 PCGs, 25–28 tRNAs, four rRNAs, and one to seven pseudogenes (Table 1). Among Loranthaceae taxa sampled in this study, the facultative root-parasite *Nuytsia floribunda* possessed a relatively larger plastome size (139,027 bp) compared to obligate stem-parasites ranging from 115,635 − 129,570 bp (Table 1), as well as a higher number of potentially functional plastid genes (98 vs.91–96) (Table 1).

**Table 1:** Comparison of size and GC content (GC%) of complete plastomes, large single copy (LSC), small single copy (SSC), and inverted repeat (IR) regions, and gene content of Loranthaceae species with the autotrophic plant *Erythropalum scandens*

Species	Plastome			LSC			IRs			SSC		Total plastid genes	Potentially functional genes	Protein encoding genes	tRNA	rRNA	Deleted genes	Pseudo -genes	
	Size (bp)	GC (%)		Size (bp)	GC (%)		Size (bp)	GC (%)		Size (bp)	GC (%)								
*Taxillus balansae*	122,438	37.3%		70,556	34.8%		22,906	42.8%		6,070	26.2%	97	94	63	27	4	17	3	
*T. thibetensis*	122,497	37.3%		70,605	34.7%		22,911	42.8%		6,070	26.2%	97	94	63	27	4	17	3	
*T. vestitus*	122,200	37.3%		70,250	34.8%		22,923	42.8%		6,104	26.2%	97	94	63	27	4	17	3	
*T. nigrans*	121,419	37.4%		70,181	34.8%		22,569	42.9%		6,100	26.2%	95	93	63	26	4	19	2	
*T. sutchuenensis*	122,589	37.3%		70,639	34.7%		22,921	42.8%		6,108	26.2%	97	94	63	27	4	17	3	
*T. pseudochinensis*	122,812	37.3%		70,878	34.6%		22,915	42.8%		6,104	26.3%	97	94	63	27	4	17	3	
*T. tsaii*	122,635	37.3%		70,703	34.7%		22,914	42.8%		6,104	26.3%	97	94	63	27	4	17	3	
*T. lonicerifolius*	122,398	37.3%		70,698	34.7%		22,822	42.9%		6,056	26.4%	97	94	63	27	4	17	3	
*T. yadoriki*	122,192	37.3%		70,628	34.6%		22,756	42.8%		6,052	26.4%	97	94	63	27	4	17	3	
*T. levinei*	122,274	37.3%		70,502	34.7%		22,844	42.9%		6,084	26.4%	97	94	63	27	4	17	3	
*T. liquidambaricola*	123,074	37.2%		71,241	34.5%		22,874	42.8%		6,085	26.4%	97	94	63	27	4	17	3	
*T. matsudae*	122,319	37.4%		70,317	34.8%		22,951	42.9%		6,100	26.1%	97	94	63	27	4	17	3	
*T. caloreas*	120,663	37.3%		69,675	34.7%		22,417	42.8%		6,154	26.1%	96	93	63	26	4	18	3	
*T. theifer*	122,126	37.3%		70,565	34.6%		22,689	43.0%		6,183	26.1%	97	94	63	27	4	17	3	
*T. sericus*	123,879	37.2%		71,723	34.5%		22,998	42.9%		6,160	26.3%	97	94	63	27	4	17	3	
*T. chinensis*	121,367	37.3%		70,359	34.7%		22,463	43.0%		6,082	26.2%	96	94	63	27	4	18	2	
*Scurrula chingii*	122,770	37.2%		70,672	34.5%		23,001	42.8%		6,096	26.1%	96	94	63	27	4	18	2	
*S. buddleioides*	122,292	37.2%		70,132	34.5%		23,056	42.8%		6,048	26.0%	96	94	63	27	4	18	2	
*S. atropurpurea*	122,457	37.2%		70,160	34.5%		23,088	42.7%		6,121	26.1%	96	94	63	27	4	18	2	
*S. parasitica*	122,561	37.2%		70,193	34.6%		23,131	42.8%		6,106	26.0%	96	94	63	27	4	18	2	
*S. pulverulenta*	119,811	37.1%		70,254	34.7%		21,741	42.4%		6,075	26.3%	94	92	63	25	4	20	2	
*S. notothixoides*	123,810	37.3%		71,448	34.7%		23,101	42.9%		6,160	26.4%	97	91	60	27	4	17	6	
*Phyllodesmis delavayi*	119,914	37.1%		70,253	34.7%		21,859	42.5%		5,943	26.9%	95	92	63	25	4	19	3	
*Helixanthera parasitica*	125,037	36.5%		73,103	33.8%		22,784	42.3%		6,366	25.5%	94	92	63	25	4	20	2	
*H. terrestris*	121,217	36.8%		70,902	34.3%		22,047	42.4%		6,221	25.3%	95	93	64	25	4	19	2	
*H. sampsonii*	120,658	36.7%		71,487	34.1%		21,495	42.6%		6,181	25.4%	95	93	64	25	4	19	2	
*Dendrophthoe pentandra*	115,635	37.0%		69,368	34.6%		20,118	42.8%		6,031	26.0%	93	92	63	25	4	21	1	
*Tolypanthus maclurei*	123,581	36.8%		72,952	34.3%		22,185	42.4%		6,259	26.0%	95	93	64	25	4	19	2	
*Helicanthes elasticus*	128,805	35.9%		76,441	33.4%		22,322	42.5%		7,719	22.5%	94	92	63	25	4	20	2	
*Plicosepalus curviflorus*	120,181	36.6%		69,498	33.9%		22,322	42.3%		6,038	24.7%	92	91	62	25	4	22	1	
*Plicosepalus acaciae*	121,086	36.8%		69,947	34.2%		22,476	42.3%		6,187	25.0%	93	92	63	25	4	21	1	
*Moquiniella rubra*	123,076	36.8%		72,597	34.3%		22,164	42.5%		6,151	26.1%	95	94	65	25	4	19	1	
*Loranthus pseudo-odoratus*	124,474	37.3%		70,550	34.8%		23,885	42.7%		6,154	25.3%	98	94	62	28	4	16	4	
*Loranthus delavayi*	125,239	37.4%		71,323	34.8%		23,866	42.7%		6,184	25.3%	98	94	62	28	4	16	4	
*L. kaoi*	124,322	37.3%		70,526	34.7%		23,865	42.7%		6,066	25.5%	98	94	62	28	4	16	4	
*L. odoratus*	121,000	37.0%		70,595	34.8%		22,140	42.2%		6,125	25.3%	96	93	63	26	4	18	3	
*L. guizhouensis*	122,419	37.2%		70,459	34.8%		22,922	42.4%		6,116	25.8%	97	94	63	27	4	17	3	
*L. lambertianus*	125,032	37.3%		70,809	34.7%		24,022	42.7%		6,179	25.2%	98	95	63	28	4	16	3	
*L. grewingkii*	122,216	37.1%		70,101	34.8%		23,081	42.2%		5,953	25.3%	97	93	62	27	4	17	4	
*L. tanakae*	121,763	37.1%		69,578	34.8%		23,075	42.2%		6,035	25.2%	97	93	62	27	4	17	4	
*L. europaeus*	120,994	37.2%		70,318	34.8%		23,007	42.2%		5,059	27.2%	96	92	62	26	4	18	4	
*Cecarria obtusifolia*	116,508	37.2%		67,873	34.7%		20,961	43.0%		6,713	25.5%	98	91	59	28	4	16	7	
*Macrosolen tricolor*	126,621	37.6%		71,895	35.2%		24,703	42.4%		5,320	25.8%	97	96	64	28	4	17	1	
*M. bibracteolatus*	127,059	37.8%		70,581	35.5%		25,446	42.3%		5,586	26.2%	97	96	64	28	4	17	1	
*M. cochinchinensis*	129,570	37.3%		73,052	34.9%		25,397	42.2%		5,724	24.1%	97	96	64	28	4	17	1	
*Elytranthe parasitica*	127,769	37.3%		71,800	34.8%		25,150	42.3%		5,669	25.4%	97	96	64	28	4	17	1	
*E. albida*	128,955	37.5%		73,092	35.0%		25,306	42.3%		5,251	26.5%	97	96	64	28	4	17	1	
*Nuytsia floribunda*	139,027	37.2%		79,519	34.8%		26,801	42.0%		5,906	25.8%	100	98	66	28	4	14	2	
*Erythropalum scandens*	156,154	38.0%		84,799	36.2%		26,394	42.8%		18,567	32.3%	114	113	79	30	4	0	1	

## Phylogenetic relationships

**Fig. 1:**
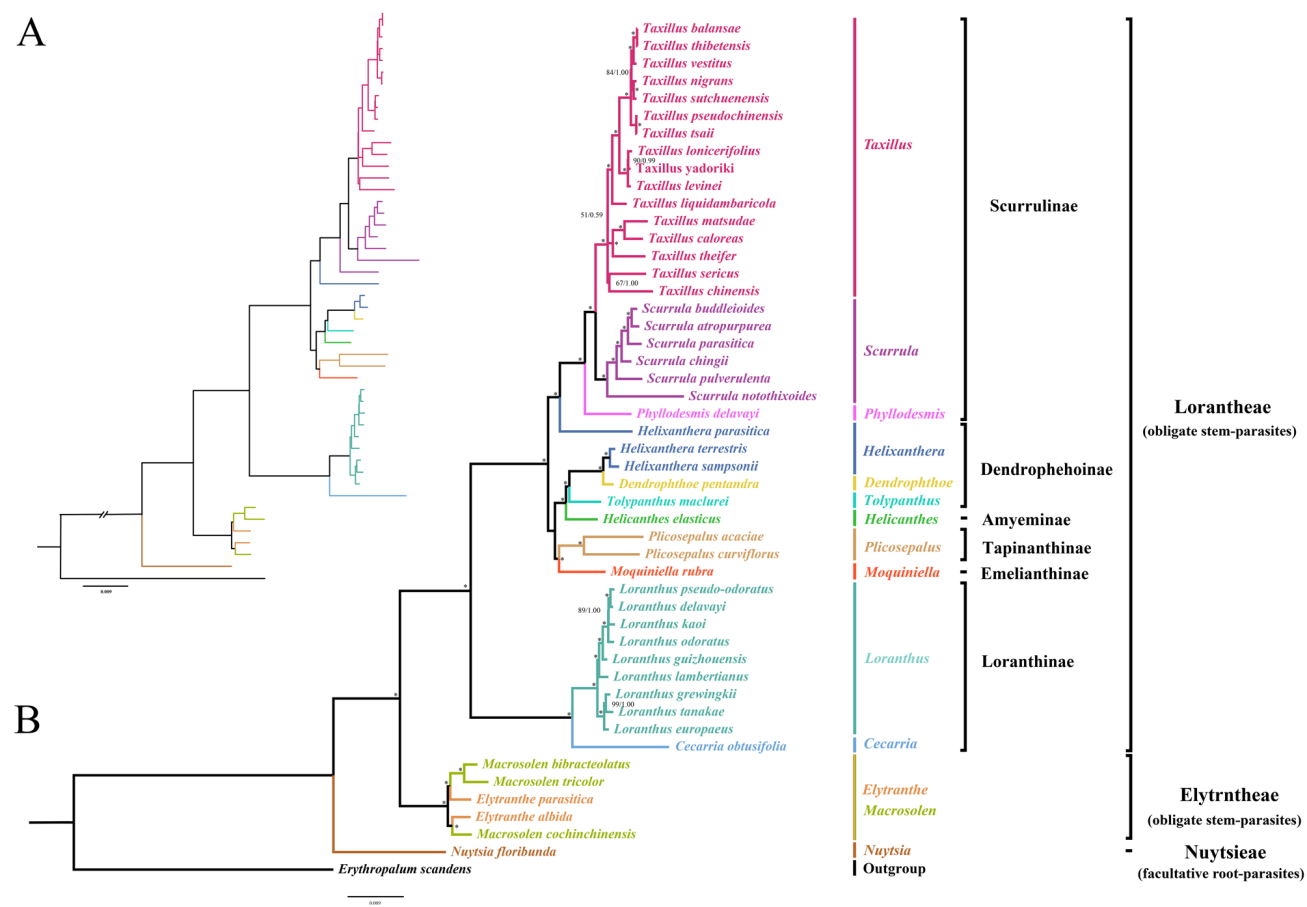
Phylogeny of Loranthaceae reconstructed by analyzing 55 plastid protein-coding genes (PCGs) using Bayesian inference (**A**) and Maximum likelihood (**B**) methods. Numbers on each node indicate bootstrap (BS) percentage /posterior probability (PP), with the asterisk (*) indicating full support in both two analyses (BS = 100; PP = 1.00).

ML and Bayesian inference BI analyses generated identical tree topologies (Fig. 1), with most nodes being fully supported (BS = 100%, PP = 1.00). Consistent with previous studies [20, 29, 30], the plastome phylogeny resolved all Loranthaceae obligate stem-parasites as a well-supported monophyletic lineage (BS = 100%, PP = 1.00), which was sister to the facultative root-parasite *Nuytsia floribunda* (tribe Nutsiea). Within the clade of obligate stem-parasites, a close relationship between the two tribes, Elytranthaea and Loranthaea was recovered (BS = 100%, PP = 1.00). For those six subtribes of the tribe Loranthaea (Scurrulinae, Dendrophthoinae, Amyeminae, Tapinanthinae, Emelianthinae, and Loranthae) sampled in this study, our phylogenetic analyses resolved them as three successively diverging branches, corresponding to (1) the subtribe Loranthae (BS = 100%, PP = 1.00), (2) Dendrophthoinae (except for *Helixanthera parasitica*) + Dendrophthoinae + Amyeminae + Tapinanthinae + Emelianthinae (BS = 100%, PP = 1.00), and (3) Scurrulinae + *Helixanthera parasitica* (BS = 100%, PP = 1.00). As the genus *Helixanthera* did not aggregate into a single branch, both ML and BI phylogenies failed to resolve the subtribe Dendrophthoinae as monophyletic. Within the tribe Elytranthaea, the monophyly of the two genera *Elytranthe* and *Macrosolen* was not supported by the plastome phylogeny.

### Phylogeny-oriented comparative analysis of Loranthaceae plastomes

**Fig. 2:**
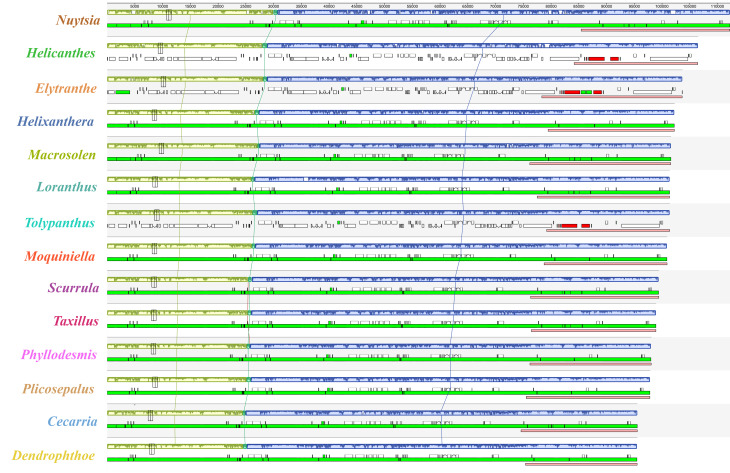
Multiple Mauve alignment of Loranthacaea plastomes

**Fig. 3:**
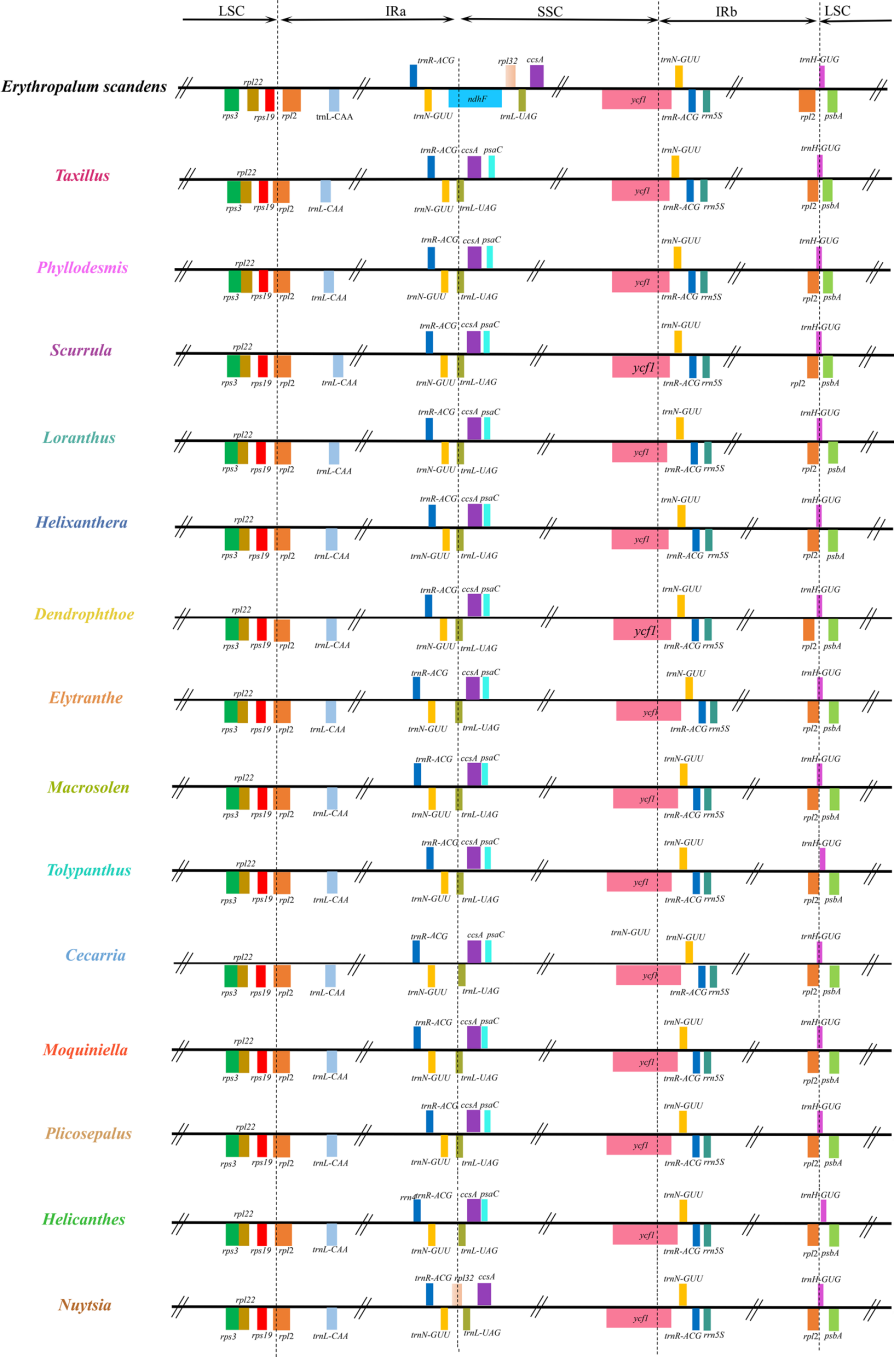
The contraction and expansion of inverted repeat regions in Loranthaceae plastomes compared with *Erythropalum scamdens* plastome

Except for a slight fragment translocation with a sequence length of 25 bp in *Phyllodemis delavayi*, microsynthetic analysis showed that no additional structural rearrangements occurred in these Loranthaceae plastomes (Fig. 2). Despite obvious variations in genome size (Table 1), these Loranthaceae plastomes exhibited a high level of conservatism with respect to their LSC/IRa, IRa/SSC, SSC/IRb, and IRb/LSC junctions (Fig. 3). Although Loranthaceae hemiparasites shared same SSC/IRb and IRb/LSC boundaries with the autotrophic relative *Erythropalum sandens*, the LSC/IRa boundaries of Loranthaceae plastomes moved into the *rpl2* gene (Fig. 3). Within Loranthaceae, the SSC/IRa boundary of the facultative root-parasite *Nuytsia floribunda* moved into the *rpl32* gene, whereas the SSC/IRa boundaries of all obligate stem-parasites were further contracted into the *trnL-UAG* gene (Fig. 3).

**Fig. 4:**
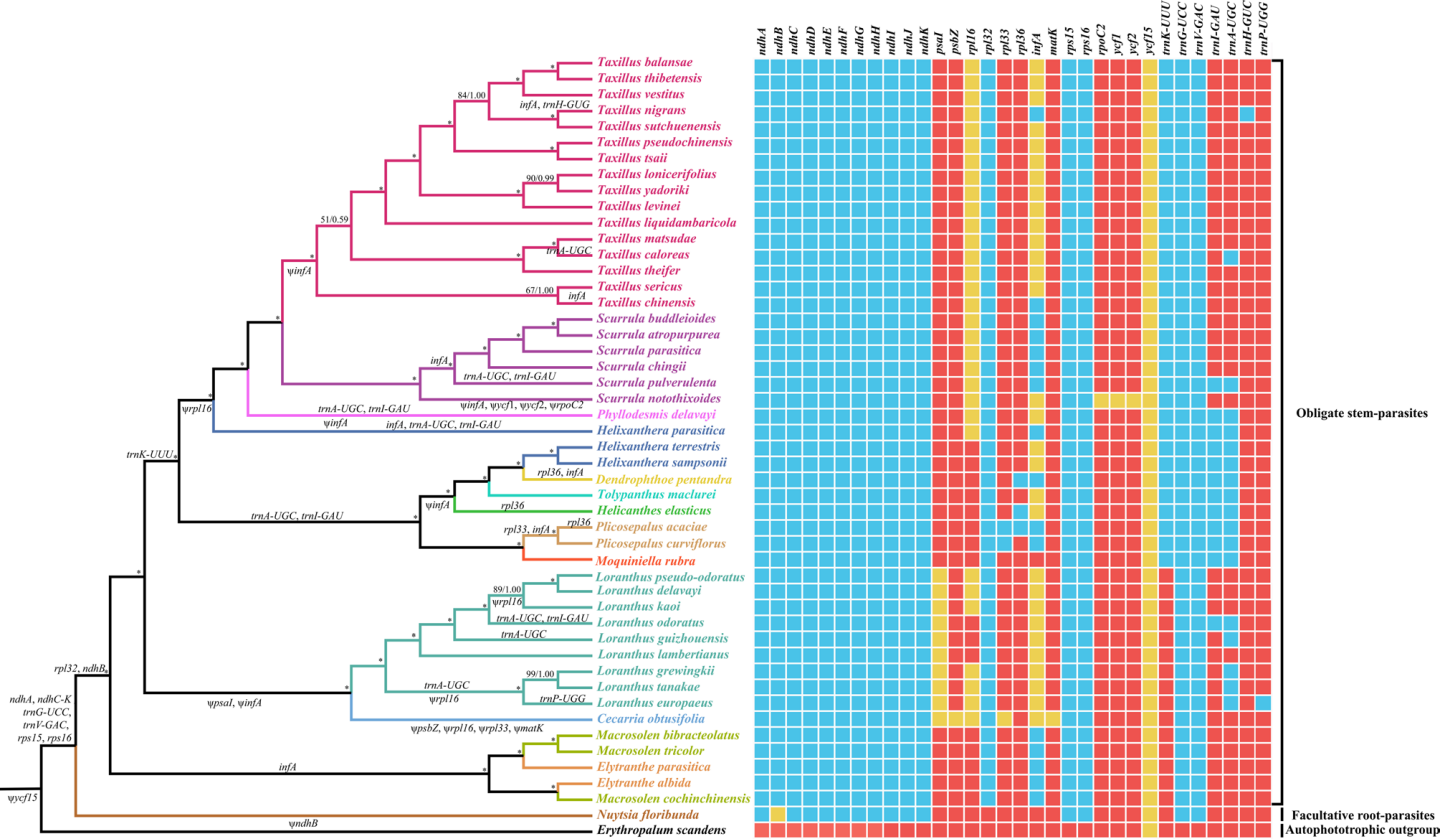
Comparison of gene content among Lorantahceae plastomes and *Erythropalum scamdens* plastome. Genes mentioned above branches indicate loss of genes, whereas under branches indicate pseudonization of genes. Red squares: intact genes; blue squares: deleted genes; yellow squares: pseudogenes

To trace the evolutionary trajectory of plastome degeneration associated with the formation of hemiparasitic lifestyle in Loranthaceae, the gene loss and pseudogenization events detected in each species were mapped to a phylogenetic tree (Fig. 4). As *ycf15* was annotated as a pseudogene in all analyzed plastomes, this mutation may have occurred in the most recent common ancestor (MRCA) of Loranthaceae and Erythropalaceae (Fig. 4). Contrary to their autotrophic relative, *Erythropalum scandens*, all plastid NADH dehydrogenase-like complex genes, except for *ndhB*, two ribosomal protein genes (*rps15* and *rps16*), and two tRNA genes (*trnG-UCC* and *trnV-UAC*), were absent from Loranthaceae plastomes, which may represent a molecular synapomorphy of the family (Fig. 4). Compared to the facultative root-parasite *Nuytsia floribunda*, additional deletion of *rpl32* and *ndhB* genes might have taken place in the stem lineage ancestor of obligate stem-parasites (Fig. 4). Among Loranthaceae obligate stem-parasites, further loss of the *infA* gene is commonly observed in the tribe Elytranthaea (Fig. 4). Within the tribe Loranthaea, the pseudogenization of *pasI* and *infA* might have occurred in the stem lineage ancestor of the subtribe Loranthinae, and the losses of *trnA-UGC* and *trnL-GAU* likely occurred in the MRCA of the subtribes Dendrophthoinae (except for *Helixanthera parasitica*), Dendrophthoinae, Amyeminae, Tapinanthinae, and Emelianthinae (Fig. 4). Furthermore, the pseudogenization of plastid *rpl16* gene could have taken place in the MRCA of the subtribes Scurrulinae and *Helixanthera parasitica* (Fig. 4).

Following the lineage-specific gene loss and pseudogenization events that occurred in the deep nodes of Loranthaceae tree topology, extensive gene loss or pseudogenization occurred independently in most shallow branches, such as the loss of *trnA-UGC* and *trnI-GAU* genes in *Tolypanthus maclurei*, *Phyllodemis delavayi*, *Scurrula pulverulenta*, *Moquiniella rubra*, *Helicanthes elasticus*, and *Loranthus odoratus* (Fig. 4). Additionally, independent deletion of *trnA-UGC* was observed in *Taxillus caloreas*, *Loranthus guizhouensis*, *Loranthus grewinghii*, *Loranthus europaeus*, and *Loranthus tankae*; independent loss of *rpl36* was detected in *Dendrophthoe pentandra*, *Helicanthes elasticus*, and *Plicosepalus acacia*; and the independent loss of *rpl33* was identified in *Plicosepalus acacia*, *Plicosepalus curviflorum*, and *Cecarria abtusifolia* (Fig. 4). Moreover, species-specific pseudogenization of *psbZ* and *matK* was detected in *Cecarria abtusifolia* (Fig. 4).

## Discussion

### Characterization of plastome degradation in Loranthaceae hemiparasites

A growing body of evidence suggests that the lifestyle transition from autotrophy to heterotrophy often results in rampant plastome degradation in parasitic plants, including the physical and functional loss of plastid genes, genome downsizing, and structural mutations [13, 14, 16, 23]. Consistent with this, the current study found that the plastomes of 48 Loranthaceae hemiparasites exhibited varying degrees of plastome size shrinkage, structural alteration, gene loss, and pseudogenization compared to their autotrophic relative, *Erythropalum scandens*. The findings indicate that although the hemiparasitic plants enable photosynthesis, their plastomes have experienced diverse modifications owing to the evolution of heterotrophic lifestyles.

Compared to their autotrophic relative, *Erythropalum scandens*, extensive gene loss and pseudogenization events were detected in the plastomes of Loranthaceae hemiparasites. Among them, *Cecarria obtusifolia* (116,508 bp), *Dendrophthoe pentandra* (115,635 bp), *Helixanthera sampsonii* (1206,58 bp), *Loranthus europaeus* (120,994 bp), *Plicosepalus curviflorus* (120,181 bp), *Scurrula delavayi* (119,914 bp), *Scurrula pulverulenta* (119,811 bp), and *Taxillus caloreas* (120,663 bp) possessed relatively smaller plastomes sizes and retained less functional plastid genes (91–93), suggesting that the plastome size shrinkage observed in Loranthaceae hemiparasites can be partially attributed to the physical or functional losses of plastid genes. Additionally, the deletion of 10 plastid *ndh* genes (*ndhA*, *ndhC–K*), two ribosomal protein genes (*rps15* and *rps16*), and two tRNAs (*trnG-UCC* and *trnV-GAC*) was observed in all Loranthaceae hemiparasites. Deletion of seven *ndh* genes (*ndhA*, *ndhD*–*I*) and rps15 in the SSC region, and three *ndh* genes (*ndhD, ndhJ*, and *ndhK*) and *trnG-UCC* in the LSC region, is respected to result in a more pronounced contraction of these two single copy regions compared to the deletion of two genes (*trnV-GAC* and *rps16*) in the IR regions. Consequently, these structural alterations lead to shifts of LSC/IRa and SSC/IRa boundaries in Loranthiaceae plastomes.

Compared to *Erythropalum scandens*, gene loss and pseudogenization events occurring in the plastomes of Loranthaceae hemiparasites involved the following PCGs: *ndhA*–*K*, *infA*, *rpl16*, *rpl32*, *rpl33*, *rpl36*, *rps15*, *rps16*, *psaI*, *psbZ*, *matK*, *ycf1*, *ycf2*, *ycf15*, and *rpoC2*, as well as six tRNAs. Among them, only three PCGs (*rps16*, *ycf2*, and *ycf15*) and four tRNAs (trnA-UCC, *trnH-GUG*, trnI-GAD, and *trnV-GAC*) were located in the IR regions, whereas the remaining genes were located in the LSC and SSC regions. Although the plastomes of Loranthiaceae hemiparasites possessed lower GC content in the LSC and SSC regions than *Erythropalum scandens*, there were slight differences in the GC content in the IR regions. The higher levels of gene loss/pseudogenization and lower GC content in the LSC and SSC regions, in contrast to the lower levels of gene loss/pseudogenization and higher GC content in the IR regions observed in the plastomes of Loranthaceae hemiparasites, provide robust evidence for the theoretical prediction that loss/pseudogenization of plastid genes in parasitic plants may occur simultaneously with a decrease in GC content [16].

Previous studies have demonstrated that parasitic plants with low GC content in their plastomes tend to accumulate inversions and structural mutations around the IR regions [13, 14]. Although varying degrees of plastid gene loss occurred in Loranthiaceae hemiparasites, significant synteny was observed among their plastomes, suggesting that they did not undergo dynamic structural rearrangement after the formation of hemiparasitic lifestyle. Notably, compared with their autotropic relative, *Erythropalum scandens* (38.0%), the GC content of Loranthaceae plastome (35.9–37.8%) was only slightly decreased. The findings provide additional empirical evidence that a dramatic decrease in GC content is more likely to trigger the structural rearrangement of the parasitic plastomes [13, 14]. Due to a higher degree of plastid gene loss and reduction in GC content observed in the plastomes of holoplastic plants compared to hemiparasitic plants, the plastomes of holoparasites are more prone to independent structural rearrangement, as previous study demonstrated [46–49].

The gene contents of Loranthaceae plastomes and their autotrophic relative were compared based on the phylogenetic tree topologies. The results indicated that a total of 10 *ndh* genes (*ndhA*, *ndhC*–K), two ribosomal protein genes (*rps15* and *rps16*), and two tRNA genes (*trnG-UCC* and *trnV-UAC*) might have been deleted in the stem lineage ancestor of Loranthaceae. The deleted *ndh* genes encode 10 subunits of the plastid NDH complex that mediates photosystem I cyclic electron transport and facilitates chlororespiration in plant cells [50]. The functional and physical loss of most plastid *ndh* genes has been commonly observed in a wide spectrum of parasitic angiosperm lineages and is regarded as an early response to the evolution of heterotrophic lifestyle [13, 14, 16]. Notably, this mutation has also been detected in a wide spectrum of photoautotrophic angiosperms [48, 51–54], and Frailey et al. proposed that the plastid *ndh* genes may have undergone negative selection in these photoautotrophic lineages [49]. Additionally, Lin et al. observed that several *ndh* genes encoded by the chloroplast and nuclear genomes may have been lost concomitantly in some epiphytic autotrophic orchids and proposed that this mutation may increase the possibility of evolving a heterotrophic lifestyle [19]. On this basis, we speculate that the massive reduction in the NDH pathway in the stem lineage ancestor of Loranthaceae might have played a pivotal role in triggering the transition from photoautotrophic to hemiparasitic lifestyles.

In addition to the loss of 10 plastid *ndh* loci, the deletion of two plastid ribosomal protein-encoding genes (*rps15* and *rps16*) was inferred to have occurred in the stem lineage ancestor of Loranthaceae, and further loss/pseudogenization of other plastid ribosomal protein-encoding genes (*rpl16*, *rpl32*, *rpl33*, and *rpl36*) was commonly observed in Loranthaceae obligate stem-parasites. Notably, loss/pseudogenization of these plastid ribosomal protein-encoding genes has been detected not only in parasitic plants [55, 56], but also in a wide range of autotrophic angiosperms [57–60]. Although the *rps15*, *rpl33*, and *rpl36* genes are not essential for chloroplast gene translation, the remaining genes play essential roles [61]. Hence, the loss of these plastid ribosomal protein-encoding genes in Loranthaceae may be compensated for by other plastid *rpl*/*rps* genes or by nuclear-encoded *rpl*/*rps* genes.

Plastid *infA* is another commonly reduced gene in the plastomes of Loranthaceae hemiparasites. While this gene was intact and retained in the facultative root-parasite *Moquiniella rubra*, the remaining Loranthaceae species (obligate stem-parasites) exhibited either loss or pseudogenization of this gene. This suggests that, after the evolution of a hemiparasitic lifestyle, plastid *infA* gene was recurrently deleted or pseudogenized in Loranthaceae obligate stem-parasites. Similarly, parallel losses of plastid *infA* genes have been observed in Santalales [24–26, 62] and a majority of holoparasitic plants [14, 16], as well as in diverse lineages of photoautotrophic angiosperms [16, 63]. Previous studies proposed that the plastid *infA* gene may have been constantly transferred to the nucleus [64]. Accordingly, horizontal transfer of the *infA* gene from plastomes to nuclear genomes may have occurred independently in Loranthaceae obligate stem-parasites.

In addition to the abovementioned protein-encoding genes, the deletion of six plastid tRNA genes was observed in the plastomes of Loranthaceae hemiparasites. It is well known that some heterotrophic plants generally contain only a fraction of tRNAs [14, 56, 65]. Despite the essential roles of tRNAs in plastid translation, the partial loss of these genes in plants may not represent a lethal mutation. Because of their relatively small size, tRNAs are easily transferred from the cytosol to plastid organelles [16], and photosynthetic plants most likely possess a specific transport mechanism that transports tRNAs to plastids from the cytosol [60, 65]. Accordingly, the loss of these tRNAs in Loranthaceae hemiparasites may not have a serious impact on their survival and is therefore tolerable.

### New insights into evolutionary trajectories of plastome degradation in hemiparasitic plants

Along with the lifestyle transition from autotrophy to heterotrophy, parasitic plants developed numerous survival strategies to adapt to various environments, which might have led to diverse plastome degradation trajectories [15–17, 56, 66]. Among hemiparasitic plants, facultative root-parasites show a slight degree of trophic reliance on the host compared to obligate stem-parasites, which require host support water and mineral elements to complete their life cycle after germination [20, 21]. Theoretically, the varying nutritional dependence of facultative root-parasites and obligate stem-parasites on their hosts may influence the reductive evolution of their plastomes [14, 22]. Although this theoretical prediction seems plausible, it has not been validated by empirical studies, leaving a critical gap in the exploration of the evolutionary trajectory of plastome degradation in hemiparasitic plants.

In the current study, the plastome phylogeny resolved all obligate stem-parasties as a well-supported monophyletic lineage sister to the facultative root-parasite, *Nuytia floribunda*, confirming the monophyletic origin of obligate stem-parasitism from facultative root-parasitism in Loranthaceae proposed by previous studies [20, 29, 30]. Compared with their autotrophic relative, *Erythropalum scandens*, the plastomes of Loranthaceae hemiparasites exhibit various degrees of plastome downsizing and gene loss/pseudogenization. Among them, the plastome size of the early diverged facultative root-parasite, *Nuytsia floribunda*, was 139,027 bp, retaining 98 functional genes, which represents the largest plastome size and highest gene content in the Loranthaceae hemiparasites sampled in this study. Contrarily, the plastomes of all obligate stem parasites possessed relatively small genome sizes (115,635–129,570 bp) and retained relatively few functional plastid genes. Comparative analyses of Loranthaceae plastomes under the phylogenetic framework also showed that the stem lineage ancestor of obligate stem-parasites might have undergone more gene deletions (losses of *rpl32* and *ndhB genes*) than the facultative root-parasite *Nuytsia floribunda* (pseudogenization of *ndhB* gene). Taken together, these results provide empirical evidence supporting the theoretical expectation that the evolution of obligate stem-parasitism from facultative root-parasitism might have caused higher levels of plastome degradation in hemiparasites [14, 22].

Nevertheless, the taxonomic sampling in this study is still subject to certain limitations. Specifically, the Loranthaceae family has been documented to include three monotypic genera (*Nuytsia*, *Atkinsonia*, and *Gaiadendron*), each hosting a facultative root-parasite [20]. Among them, *Gaiadendron* exhibits a closer phylogenetic relationship with the clade of obligate stem-parasites compared to *Nuytsia* and *Atkinsonia* [29]. Given that this study only included one facultative root-parasite (*Nuytia floribunda*), comparing its plastome with those of obligate stem-parasites may not provide an accurate reflection of the evolutionary trajectory of plastome degradation associated with the lifestyle transition in hemiparasites. From this perspective, our data may not provide robust support for the theoretical expectation that the transition from facultative root-parasitism to obligate stem-parasitism could lead to increased levels of plastome degradation in hemiparasites [14, 22]. Therefore, the further validation is necessary to determine whether the lifestyle transitions resulting in increased nutritional reliance on host plants constitute pivotal evolutionary events that trigger the ‘domino effect’ in plastome degradation of hemiparasitic plants.

Within the clade of obligate stem-parasites, only one gene (*infA*), was lost in the plastomes of the early divergent tribe Elytranthaea. Within the tribe Loranthaea, further lineage-specific gene loss and pseudogenization occurred in the stem lineage ancestors of the subtribe Loranthinae, the MRCA of the subtribes Dendrophthoinae (except for *Helixanthera parasitica*), Dendrophthoinae, Amyeminae, Tapinanthinae, and Emelianthinae, as well as the MRCA of the tribes Scurrulinae and *Helixanthera parasitica.* Additionally, independent gene loss and pseudogenization were observed in some shallow nodes of the Loranthaea tribe. As a result, taxa within shallow branches exhibit a heightened degree of physical and functional loss of plastid genes compared to taxa within deep clades (e.g., the tribe Elytranthaea). This suggests that plastome degradation in Loranthaceae hemiparasites is likely a gradual process that intensifies with lineage and species divergence. Considering the occurrence of lineage-specific gene loss/pseudogenization at deep nodes and additional independent genes loss/pseudogenization in shallow branches, it is plausible to hypothesize that cladogenesis may act as an essential evolutionary force triggering the cascading degradation (the ‘domino effect’) of plastome degradation in hemiparasitic plants.

### Phylogenetic implications

The infra-familial relationships of Loranthaceae recovered in this study were largely congruent with previous studies [20, 29, 30, 67–69] but received full support (BS = 100; PP = 1.00) for most nodes. Notably, the plastome phylogeny failed to resolve the genus *Helixanthera* as monophyletic, given that *Helixanthera parasitica* formed a sister relationship with the subtribe Scurrulinae clade, which is consistent with the findings of Su et al. [70] and Liu et al. [29]. This relationship is supported by morphological evidence that the flowers of *Helixanthera parasitica* and the two genera (*Scurrula* and *Taxillus*) of the subtribe Scurrulinae are 5-merous, which is different from the 4-merous flowers of *Helixanthera terrestris* and *Helixanthera sampsonii* [29]. Collectively, this line of evidence suggests that the current taxonomic delineation of the genus *Helixanthera* is likely problematic. To reasonably resolve this taxonomic issue, further studies based on multidisciplinary methods are required.

Consistent with a previous study [29], the plastome phylogeny showed that the two genera (*Elytranthe* and *Macrosolen*) of the tribe Elytranthaea were not monophyletic, as the species of the two genera were interwoven in the tree topology. Notably, both genera share similar vegetation, flower, and fruit morphology, and the diagnostic characteristics used to discriminate between them is that the inflorescences of *Macrosolen* have relatively larger bracts. Accordingly, Barlow argued that this trait may not be robust enough to distinguish these two genera from each other and proposed treating *Macrosolen* and *Elytranthe* as congeneric [71]. The current study provides a phylogenomic evidence supporting this taxonomic proposal.

## Conclusion

The plastome features of 48 Loranthaceae hemiparasites were characterized in this study. Under a phylogenetic framework, comparative analyses of these Loranthaceae plastomes revealed that all obligate stem-parasites exhibited a higher degree of plastid degeneration than the closely related facultative root-parasite, *Nuytia floribunda*, providing empirical evidence for the theoretical expectation that the evolution of obligate stem-parasitism from facultative root-parasitism may have caused a higher level of plastome degradation in hemiparasites [14, 22]. Phylogeny-oriented comparative analyses of Loranthaceae plastomes have also revealed that plastome degradation in Loranthaceae is likely a gradual process that intensifies with lineage and species divergence. Therefore, cladogenesis can be a key evolutionary force that triggered the ‘domino effect’ in plastome degradation of hemiparasitic plants. These findings provide new insights into the evolutionary trajectory of plastome degradation in hemiparasitic plants.

### Electronic supplementary material

Below is the link to the electronic supplementary material.

Supplementary Material 1

## Data Availability

The complete plastome sequences generated in this study can be accessed at the NCBI GenBank database, by searching their corresponding accession numbers (OR909691, OR909692, OR909704, OR909697, OR909702, OR909693, OR909707, OR909710, OR909711, OR909708, OR909709, OR909701, OR909703, OR909705, OR909695, OR909690, OR909699, OR909706, OR909700, OR909698, OR909694, OR909696).

## Abbreviations

bp: Base Pair
BS: Bootstrap
CTAB: Cetyl Trimethylammonium Bromide
DNA: Deoxyribonucleic Acid
GbP: Giga Base Pairs
GC: Guanine and Cytosine
IR: Inverted Repeat
LSC: Large Single Copy
MCMC: Markov Chain Monte Carlo
ML: Maximum Likelihood
NCBI: National Center for Biotechnology Information
PCGs: Protein-Coding Genes
PP: Posterior Probability
rRNA: Ribosomal RNA
SSC: Small Single Copy
tRNA: Transfer RNA
