# Supplementary material for: Comparative and phylogenetic analyses of Loranthaceae plastomes provide insights into the evolutionary trajectories of plastome degradation in hemiparasitic plants
Source: BMC Plant Biol. 2024 May 16;24:406. doi: 10.1186/s12870-024-05094-5 (PMC11097404; doi:10.1186/s12870-024-05094-5)
Supplement: Supplementary file 1 — Supplementary Material 1 [file 12870_2024_5094_MOESM1_ESM.docx]

**Table S1.** Voucher information and GenBank accession numbers of the 22 Loranthaceae species newly sequenced in this study.

| Taxa | Locality | Voucher* | GenBank Accessions |
| --- | --- | --- | --- |
|  |  |  | Complete plastome |
| *Taxillus balansae* | Myanmar | DM20771 | OR909691 |
| *Taxillus thibetensis* | Myanmar | DM20845 | OR909692 |
| *Taxillus sutchuenensis* | Wenshan, Yunnan, China | DC1 | OR909704 |
| *Taxillus levinei* | Kunming, Yunnan, China | DM24504 | OR909697 |
| *Taxillus caloreas* | Nujiang, Yunnan, China | 10CS2171 | OR909702 |
| *Taxillus sericus* | Dehong, Yunnan, China | DM22354 | OR909693 |
| *Taxillus chinensis* | Guangxi, China | DM1014 | OR909707 |
| *Scurrula buddleioides* | Linzhi, Xizang (Tibet), China | DM23607 | OR909710 |
| *Scurrula atropurpurea* | Kunming, Yunnan, China | TLL054 | OR909711 |
| *Scurrula parasitica* | Changjiang, Hainan, China | DM2202 | OR909708 |
| *Scurrula chingii* | Xishuangbanna, Yunnan, China | DM1986 | OR909709 |
| *Scurrula pulverulenta* | Gengma, Yunnan, China | 13CS5903 | OR909701 |
| *Taxillus delavayi* | Shangri-la, Yunnna, China | 11CS3147 | OR909703 |
| *Helixanthera parasitica* | Rongan, Guangxi, China | DM1013 | OR909705 |
| *Helixanthera terrestris* | Linzhi, Xizang (Tibet), China | DM23441 | OR909695 |
| *Helixanthera sampsonii* | Malipo, Yunnna, China | DM5896 | OR909690 |
| *Dendrophthoe pentandra* | Baoshan, Yunnan, China | 12CS5093 | OR909699 |
| *Loranthus delavayi* | Puer, Yunnan, China | DM2078 | OR909706 |
| *Loranthus tanakae* | Baoji, Shanxi, China | TianXH577 | OR909700 |
| *Macrosolen bibracteolatus* | Honghe, Yunnan, China | DM24665 | OR909698 |
| *Elytranthe albida* | Dehong, Yunnan, China | DM22415 | OR909694 |
| *Elytranthe parasitica* | Linzhi, Xizang (Tibet), China | DM23606 | OR909696 |

**Table S2.** Publicly available complete plastomes obtained from NCBI GenBank database.

| Samples | Genbank accession |
| --- | --- |
|  | Plastome |
| *Taxillus vestitus* | MN175257 |
| *Taxillus nigrans* | MH095982 |
| *Taxillus pseudochinensis* | MW598493 |
| *Taxillus tsaii* | MW598497 |
| *Taxillus lonicerifolius* | MW598503 |
| *Taxillus yadoriki* | NC_051553 |
| *Taxillus liquidambaricola* | MW598494 |
| *Taxillus matsudae* | NC_058842 |
| *Taxillus theifer* | MW598505 |
| *Scurrula notothixoides* | MH220878 |
| *Tolypanthus maclurei* | MH922027 |
| *Helicanthes elasticus* | NC_072103 |
| *Helicanthes elasticus* | NC_072103 |
| *Plicosepalus acaciae* | NC_068660 |
| *Plicosepalus curviflorus* | NC_068661 |
| *Moquiniella rubra* | NC_058868 |
| *Loranthus pseudo-odoratus* | MT987635 |
| *Loranthus kaoi* | MT987633 |
| *Loranthus odoratus* | NC_058866 |
| *Loranthus guizhouensis* | MT987632 |
| *Loranthus lambertianus* | MT987634 |
| *Loranthus grewingkii* | NC_058861 |
| *Loranthus europaeus* | NC_058860 |
| *Cecarria obtusifolia* | MT987627 |
| *Macrosolen tricolor* | MH161425 |
| *Macrosolen cochinchinensis* | MH161424 |
| *Nuytsia floribunda* | MT987640 |
| *Erythropalum scandens* | NC_036759 |

**Table S3.** Summary of Illumina sequencing and plastome assembly.

| Taxon | No. of clean reads (bp) | Plastome | |  |
| --- | --- | --- | --- | --- |
|  |  | Size of plastid genome (bp) | No. of mapped reads (bp) | Coverage (×) |
| *Taxillus balansae* (DM20771) | 29,981,634 | 122,438 | 1,047,601 | 1288.544 |
| *Taxillus thibetensis* (DM20845) | 30,508,528 | 122,497 | 1,731,466 | 2128.162 |
| *Taxillus sutchuenensis* (DC1) | 29,786,120 | 122,589 | 1,028,181 | 1247.866 |
| *Taxillus levinei* (DM24504) | 31,463,194 | 122,274 | 3,432,628 | 4228.394 |
| *Taxillus caloreas* (10CS2171) | 11,098,268 | 120,663 | 217,575 | 259.655 |
| *Taxillus sericus* (DM22354) | 30,148,286 | 123,879 | 1,331,929 | 1621.216 |
| *Taxillus chinensis* (DM1014) | 29,555,344 | 121,367 | 1,310,486 | 1608.327 |
| *Scurrula buddleioides* (DM23607) | 27,084,530 | 122,292 | 1,219,889 | 1483.657 |
| *Scurrula atropurpurea* (TLL054) | 30,666,848 | 122,457 | 510,520 | 625.927 |
| *Scurrula parasitica* (DM2202) | 32,420,470 | 122,561 | 1,671,215 | 2032.589 |
| *Scurrula chingii* (DM1986) | 30,940,834 | 122,770 | 106,520 | 124.556 |
| *Scurrula pulverulenta* (13CS5903) | 21,553,258 | 119,811 | 2,599,928 | 3271.823 |
| *Phyllodesmis delavayi* (11CS3147) | 17,623,356 | 119,914 | 434,039 | 521.220 |
| *Helixanthera parasitica* (DM1013) | 28,609,580 | 125,037 | 332,706 | 393.859 |
| *Helixanthera terrestris* (DM23441) | 30,826,670 | 121,217 | 113,415 | 140.019 |
| *Helixanthera sampsonii* (DM5896) | 30,988,798 | 120,658 | 283,639 | 351.824 |
| *Dendrophthoe pentandra* (12CS5093) | 17,951,834 | 115,635 | 171,518 | 223.974 |
| *Loranthus delavayi* (DM2078) | 29,786,120 | 125,239 | 1,028,181 | 1247.866 |
| *Loranthus tanakae* (TianXH577) | 20,071,932 | 121,763 | 786,651 | 975.537 |
| *Macrosolen bibracteolatus* (DM24665) | 30,986,410 | 127,059 | 134,459 | 158.596 |
| *Elytranthe parasitica* (DM23606) | 31,123,496 | 127,769 | 99,436 | 115.879 |
| *Elytranthe albida* (DM22415) | 476,928 | 128,955 | 294,730 | 343.117 |


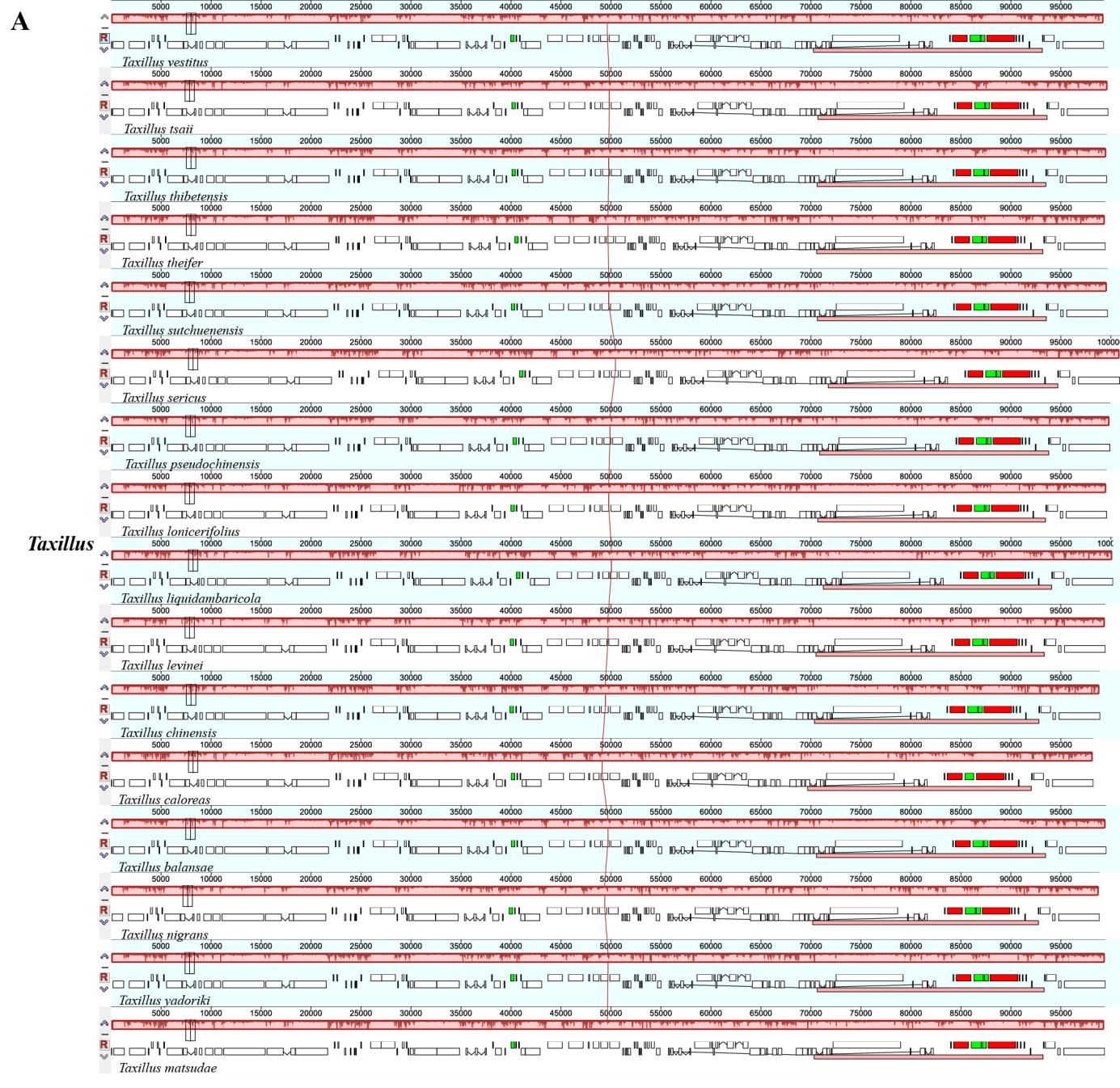


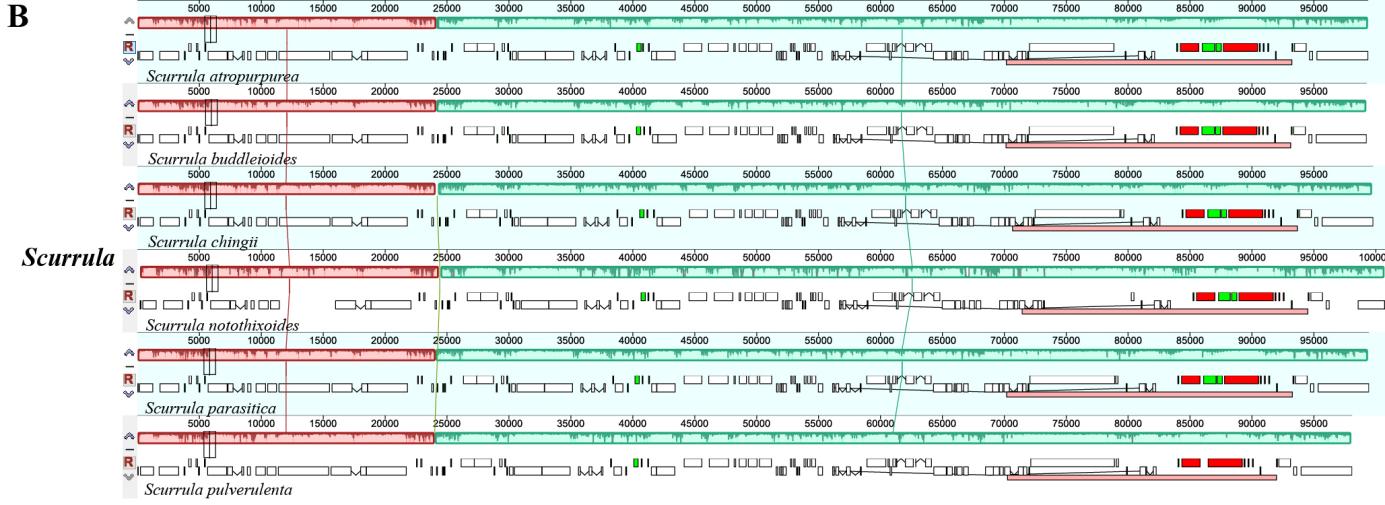


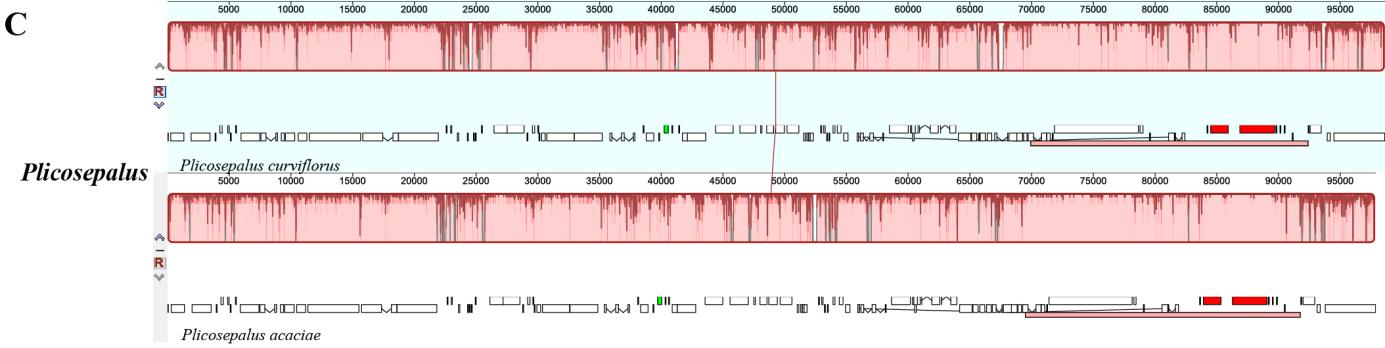


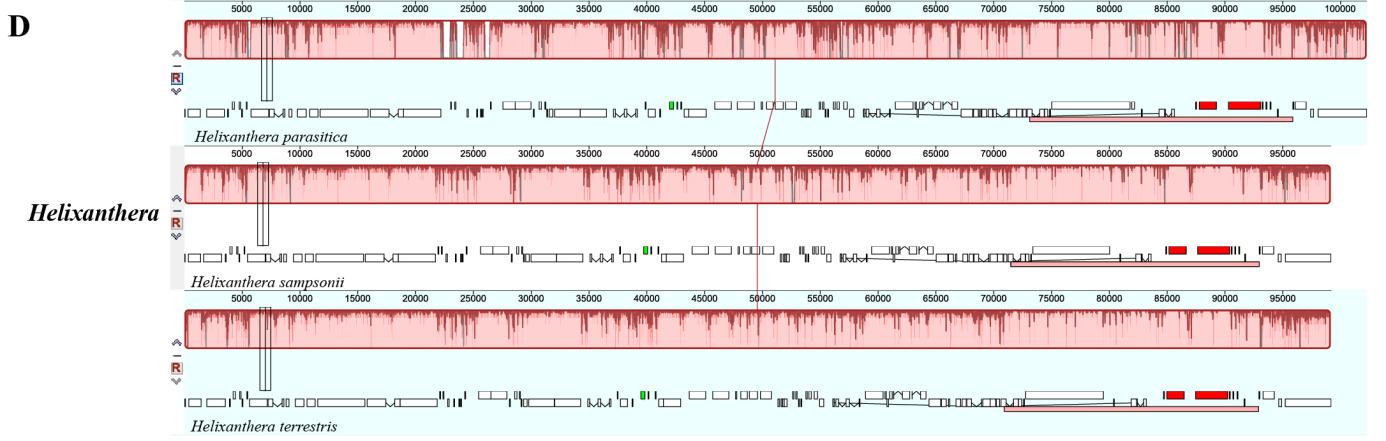


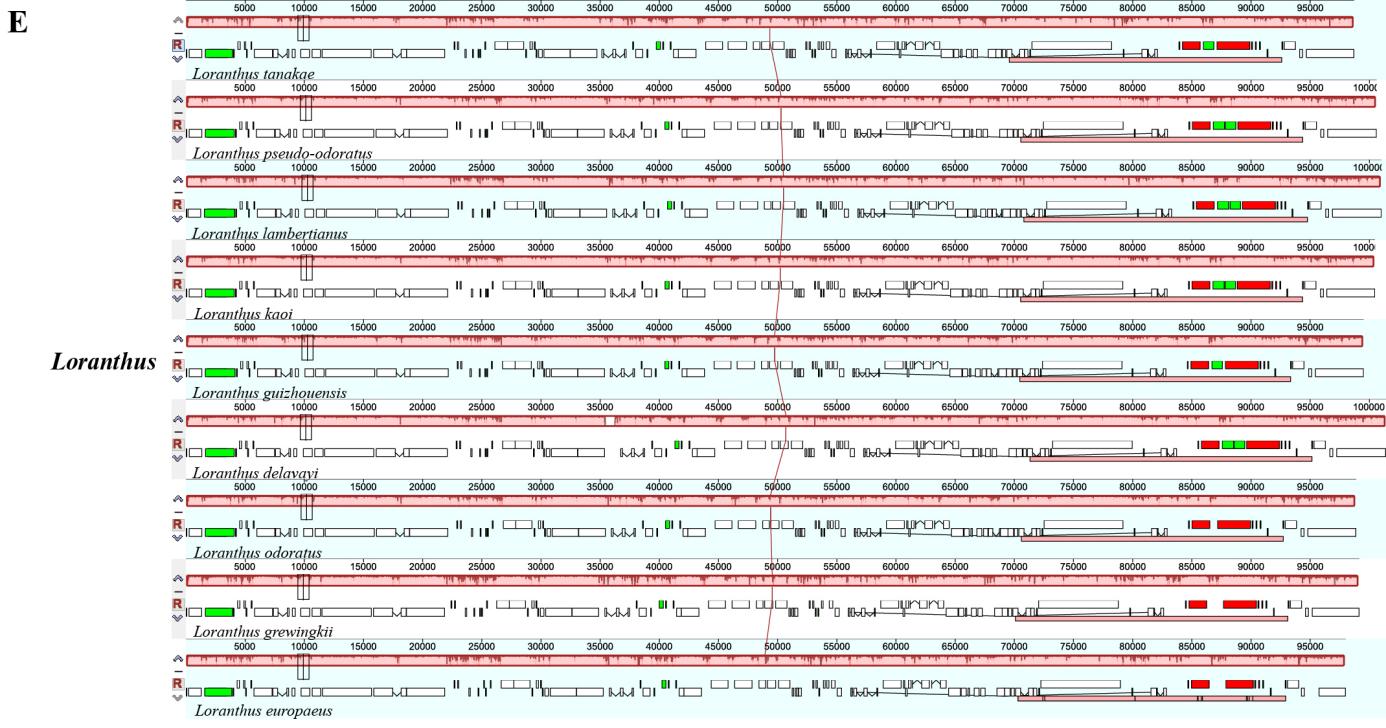


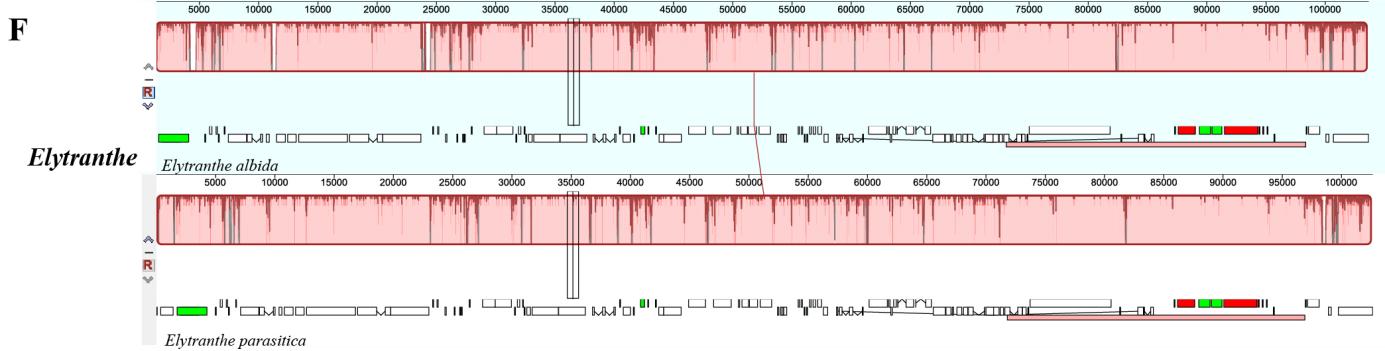


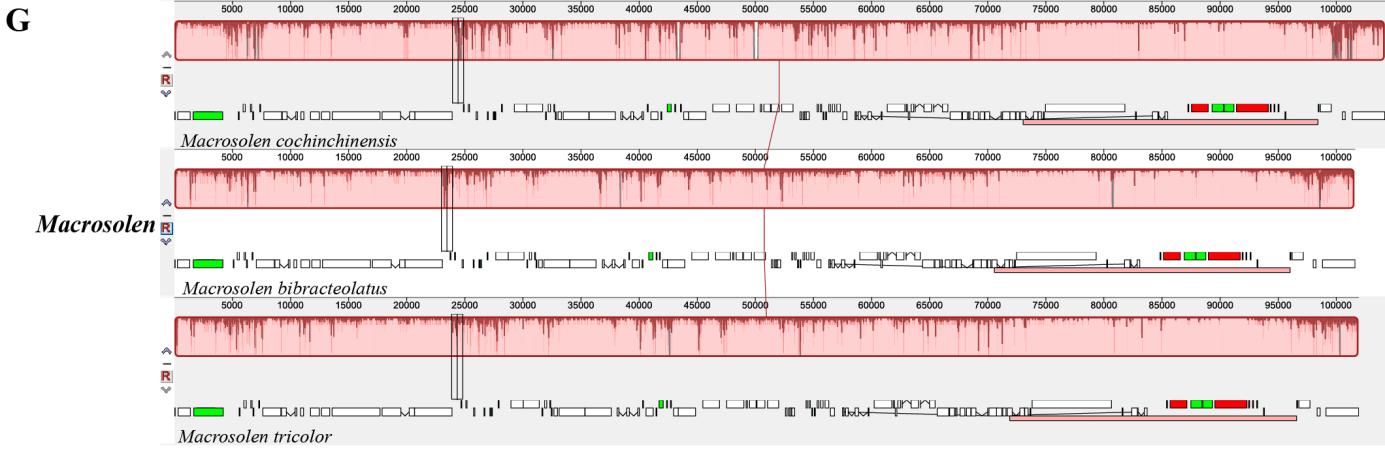


**Figure S1.** Mauve alignment of seven Lorantahceae genus: *Taxillus* (A), *Scurrula* (B), *Picosepalu* (C), *Helixanthera* (D), *Loranthus* (E), *Elytranthe* (F), and *Macrosolen* (G). Pink squares indicate the IR region.


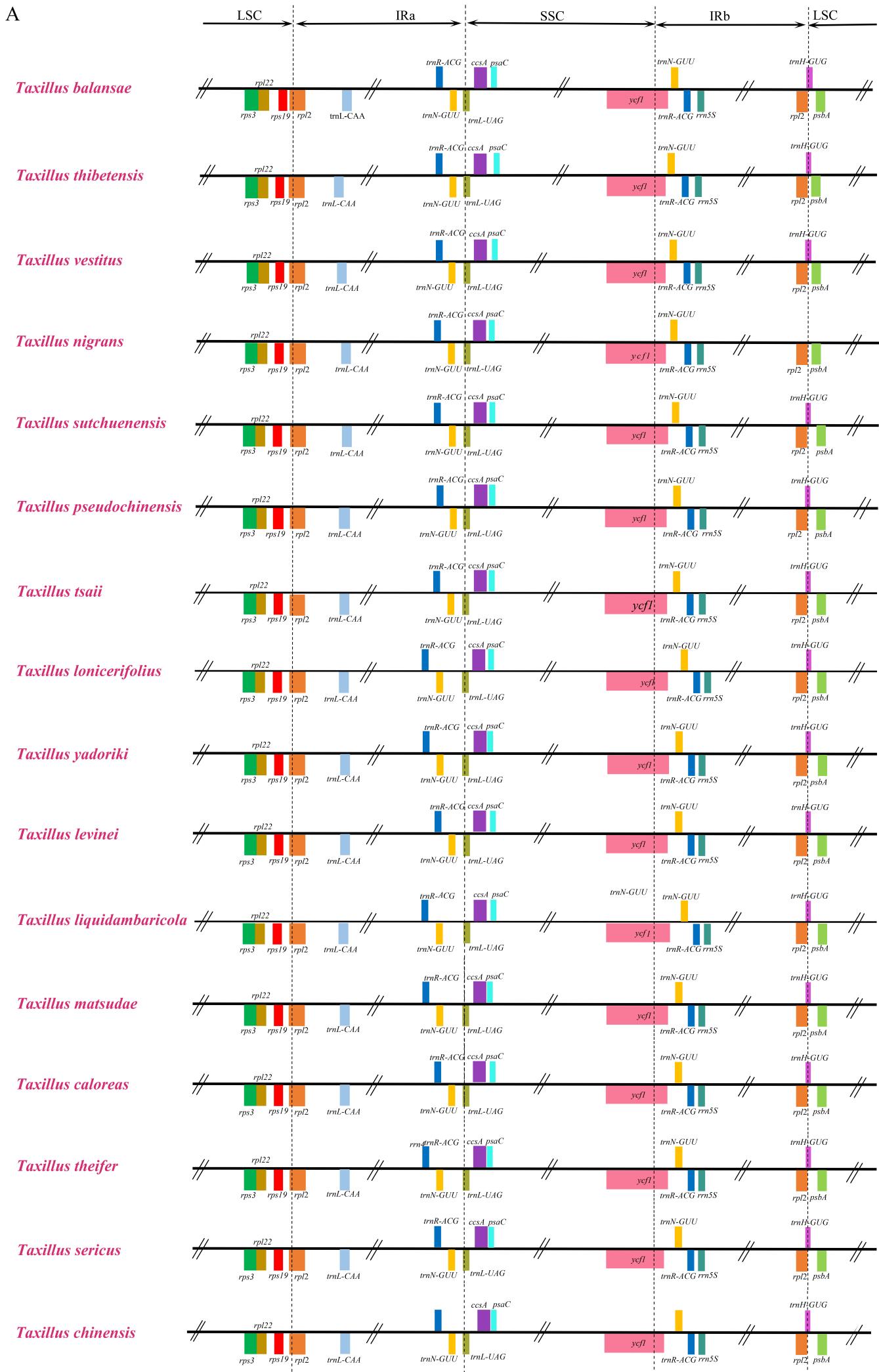


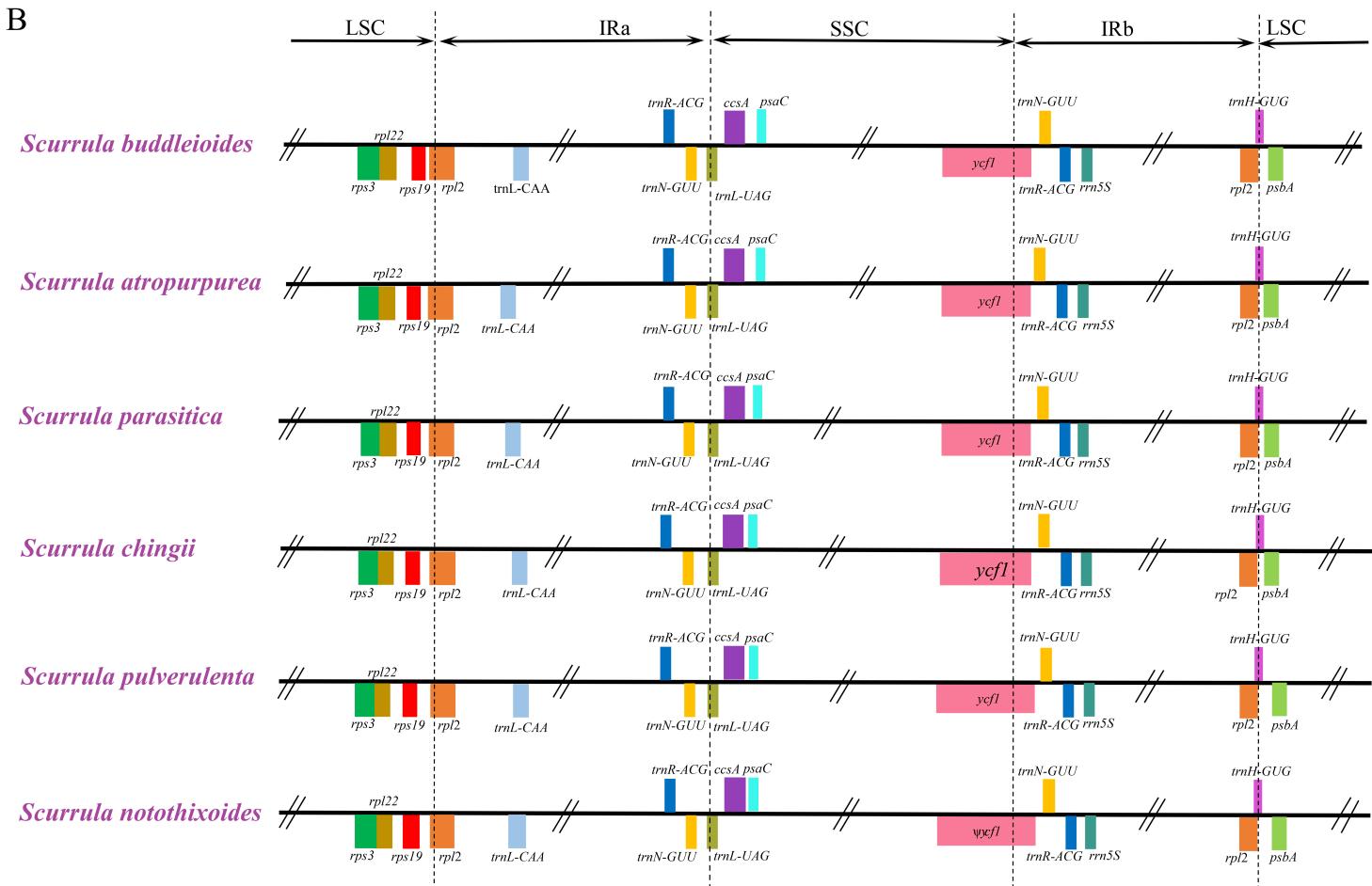


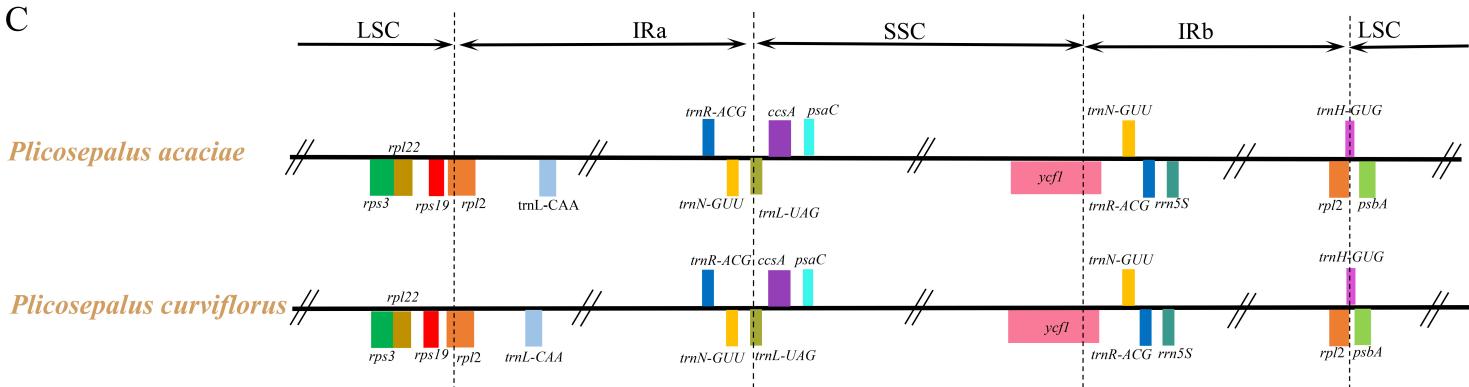


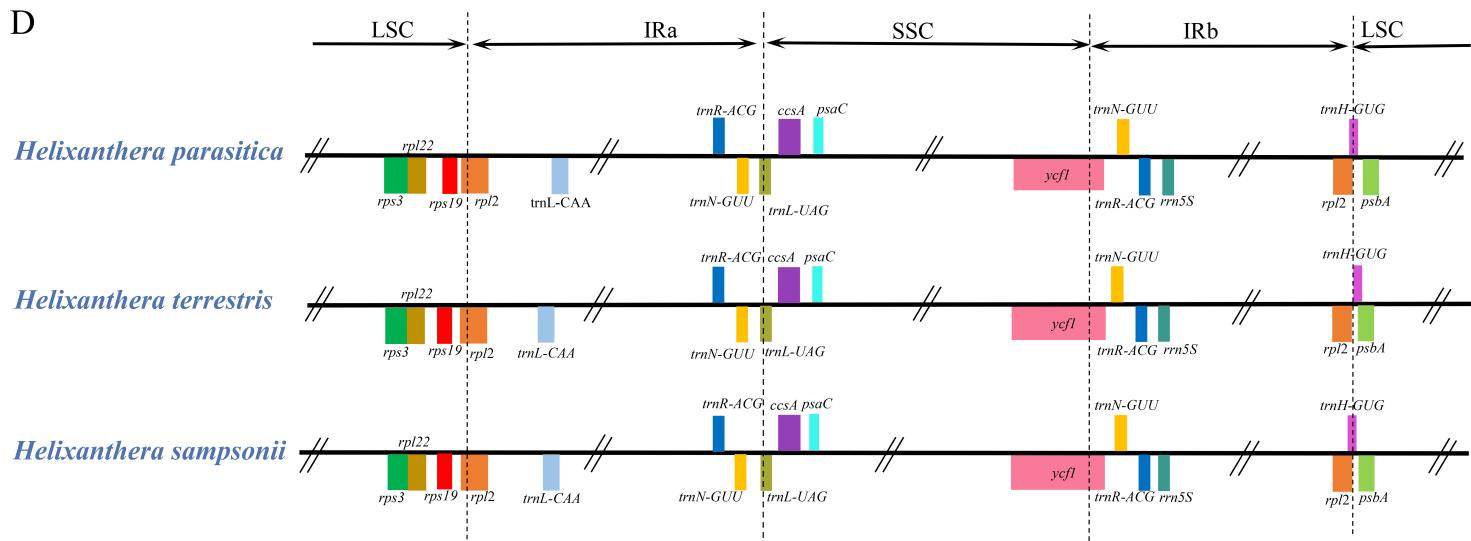


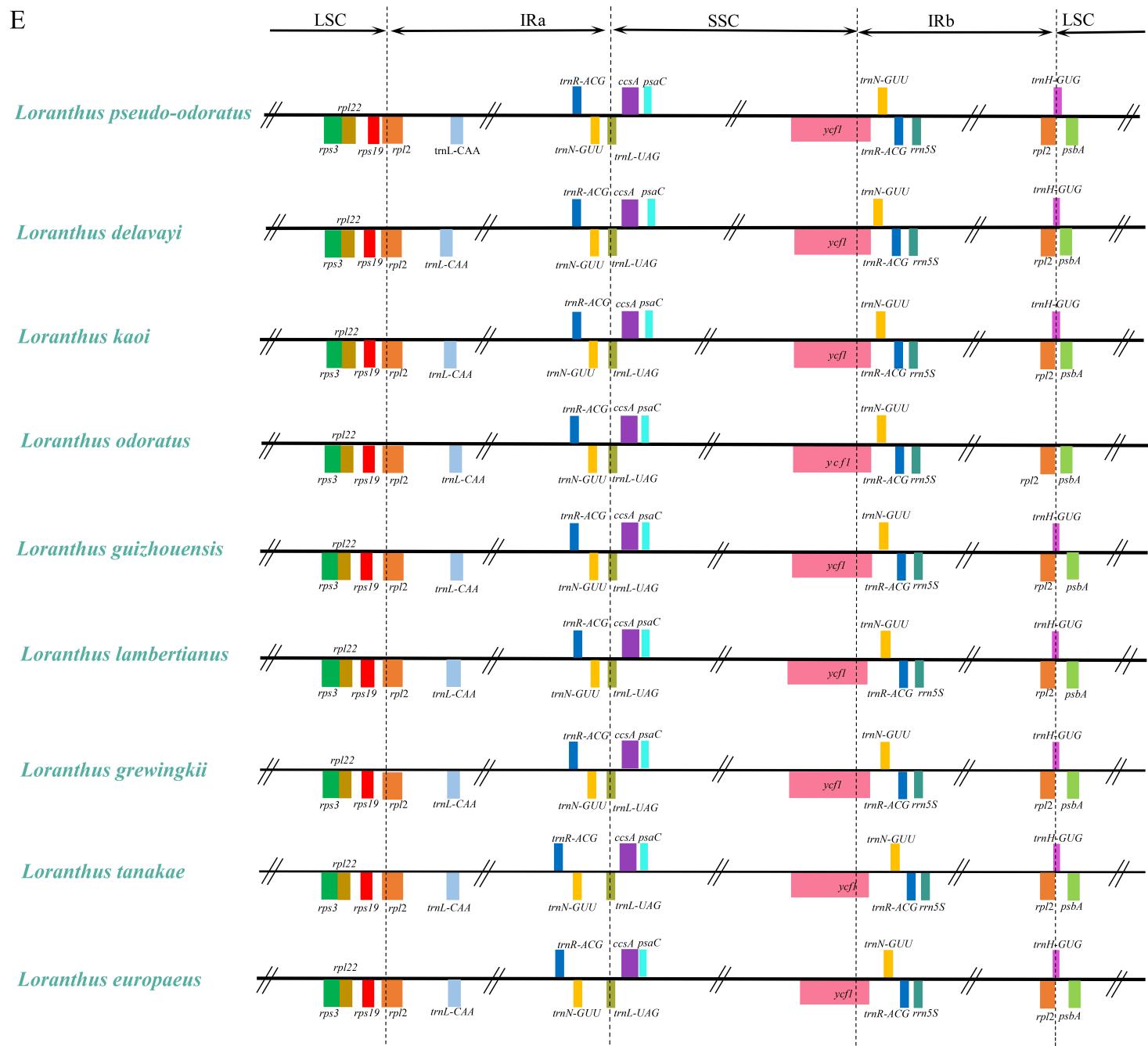


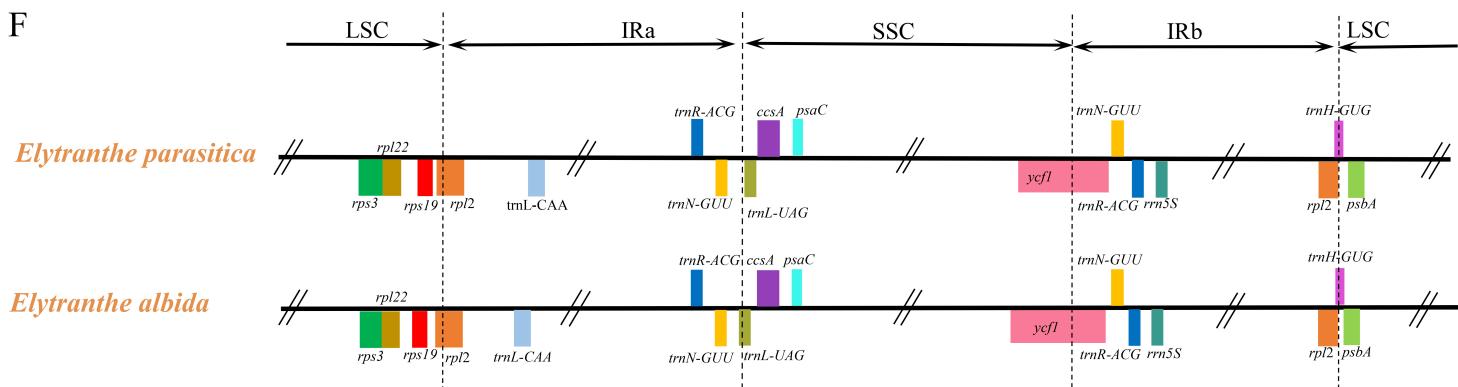


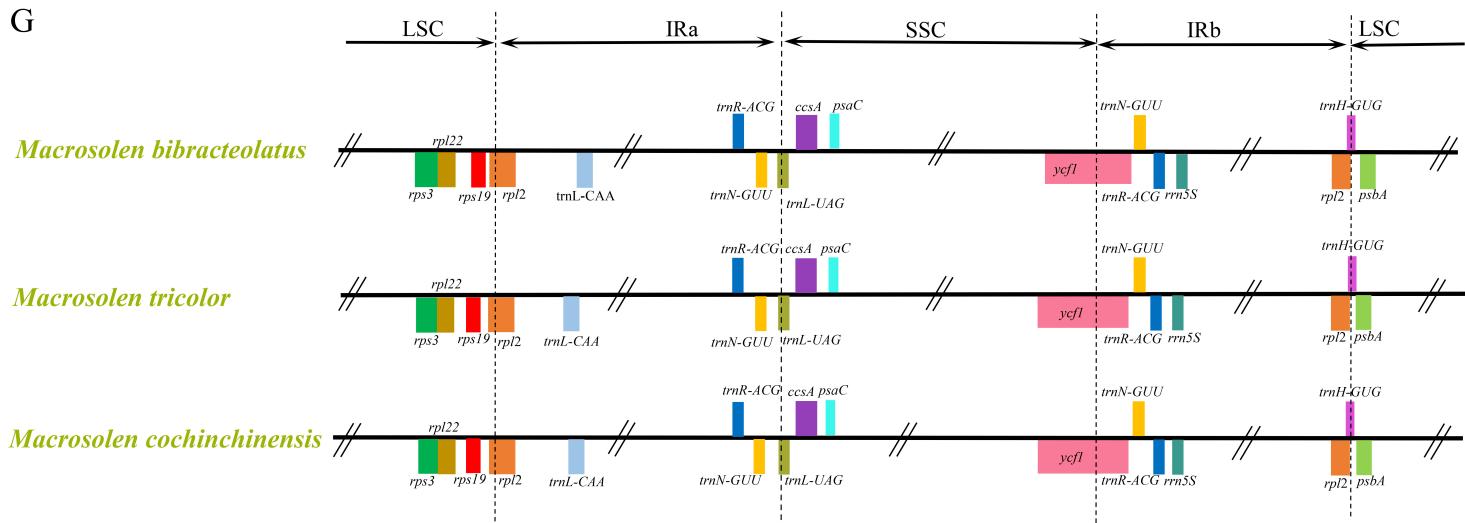


**Figure S2.** Comparison of platomes junction regions within genus: *Taxillus* (A), *Scurrula* (B), *Picosepalu* (C), *Helixanthera* (D), *Loranthus* (E), *Elytranthe* (F), and *Macrosolen* (G).
